# Vitamin E and Selenium Treatment Alleviates Saline Environment-Induced Oxidative Stress through Enhanced Antioxidants and Growth Performance in Suckling Kids of Beetal Goats

**DOI:** 10.1155/2020/4960507

**Published:** 2020-10-07

**Authors:** Nasir Mahmood, Amjad Hameed, Tarique Hussain

**Affiliations:** ^1^Animal Sciences Division, Nuclear Institute for Agriculture and Biology College, Pakistan Institute of Engineering and Applied Sciences (NIAB-C, PIEAS), Jhang Road, Faisalabad 38000, Pakistan; ^2^Nuclear Institute for Agriculture and Biology College, Pakistan Institute of Engineering and Applied Sciences (NIAB-C, PIEAS), Jhang Road, Faisalabad 38000, Pakistan

## Abstract

Salinity is a worldwide, threatening problem affecting socioeconomic status globally. Saline land comprises salt content in soil, plants, and drinking water. Livestock farming is the worthy option for proper utilization of saline land in a cost-effective approach. Animals reared on this land experience a variety of stresses. Such stresses promote oxidative stress and reduced animal performance. The purpose of this study was to investigate the antioxidative function of vitamin E and selenium (Se) on pregnant/nonpregnant animals reared on the saline environment. A total of 36 multiparous pregnant (*n* = 18) and nonpregnant (*n* = 18) goats weighing about 38-45 (average 41.5) kg were equally divided into control and supplemented groups. The experiment lasted from 120 days of gestation to 15 days after parturition for pregnant goats and 0 to 45 days for nonpregnant cyclic goats (>50 days post-kidding). The supplemented group was administered vitamin E (1000 mg/kg BW) and selenium (3 mg/50 kg BW), while the control group was kept on normal saline (0.9% NaCl) with the same route 15 days apart. The blood samples were collected with 15 days apart during the entire experimental period of 45 days and subjected to assessment of enzymatic/nonenzymatic antioxidants, hydrolytic enzymes, oxidants, stress metabolic biomarkers, Se, and progesterone concentration of (pregnant) animals. Results revealed that vitamin E and Se supplementation significantly enhanced the activity of enzymatic (catalase and peroxidase) and nonenzymatic antioxidants such as total phenolic/flavonoid content and vitamin C and increased blood plasma level of Se concentration in comparison with the control group (*P* < 0.01). Exposure to antioxidant supplementation mitigated lipid peroxidation and enhanced progesterone level and total antioxidant capacity (*P* < 0.01) as compared to the control group in pregnant goats. Administration of vitamin E and selenium promoted kid survival rate (100%) along with increased initial birth weight, daily average weight gain, and total weight gain in comparison with the control group. Besides, the twinning rate and sex ratio were also recorded in pregnant animals. It is concluded that vitamin E and Se supplementation ameliorated salinity-induced oxidative stress, improved antioxidant status, and enhanced reproductive and growth performance of suckling kids reared on saline land.

## 1. Introduction

Salinity is a worldwide problem, which affects the endogenous resources of the country and makes the saline land unprofitable. The saline land contains high level of soluble salts in the soil. Likewise, plants raised on salt-affected land have high salt content. Reclamation of saline land is very difficult, needs proper tools, and may give returns to the economy. Livestock farming is the best option for proper utilization of salt-affected land by animal farming systems [1]. Goats can easily be grown with minimum efforts without a large investment, and they can provide maximum output through milk, meat, and wool and support poor farmers localized in that area [2]. Small ruminants are very important animals due to several reasons like short generation interval, high twining and triplet ratio, and accelerated growth and can easily be adopted on low-quality forages [3]. The major problem for animals in such land is the prolonged intake of salts in terms of drinking water and salt-affected plants which display deleterious effects and even cause the death of the animals if proper attention has not paid [4]. Plants of high salt content consumed by animals may disrupt animal physiology and eventually influence the reproductive efficiency of the animals. Another potential impact of rearing animals on saline land is that these animals experience the variety of saline stresses which results in an overwhelming amount of reactive oxygen species (ROS) due to depletion of the antioxidant system [5, 6]. High level of salt intake alters the cellular redox homeostasis via promoting the activity of nicotinamide adenine dinucleotide phosphate hydrogen (NADPH) oxidase and suppresses the expression of some enzymatic antioxidants such as superoxide dismutase (SOD), catalase (CAT), and glutathione peroxidase (GPX) [7].

The most important ROS includes hydroxyl radicals, superoxide anions, and hydrogen peroxide; these are the by-products of oxidative metabolism. Some other sources of ROS are NADPH oxidase and xanthine oxidase. The antioxidants such as enzymatic (SOD, CAT, and GPX) and nonenzymatic (such as GSH, Vit E, ascorbic acid, and selenium) both display detoxifying function against oxidants [8]. Physiologically active ROS plays a pivotal role in cell signaling, gene expression, protein expression, intrinsic apoptosis, etc. to stabilize the cellular environment [9]. Once the balance of the oxidant and the antioxidant system is severely disturbed due to overproduction of oxidative stress, it may lead to damaging biological molecules like lipid membranes, cellular organelles and proteins, and nuclear deoxyribonucleic acid/mitochondrial deoxyribonucleic acid (nDNA/mtDNA) and eventually leads to causing cellular death (apoptosis) [10].

Gestation is a natural phenomenon in which the metabolic rate accelerates to fulfill the energy requirements of the mother as well as the growing fetus in the womb [11]. As the pregnancy advances, oxygen requirement and energetic metabolism upregulate which puts pressure on mitochondria to produce more energy, thus leading to oxidative stress [11]. Moreover, higher intake of salts during pregnancy may disturb the redox balance [4]. It is well documented that oxidative stress confers a predisposing factor for different pregnancy disorders like fetal growth restriction, stillbirth, and retained placenta [9]. Offering antioxidant supplementation during the last trimester of pregnancy prevents animals from the deleterious effects of oxidative stress and improves animal health [12]. Vitamin E and Se are considered crucial nutrients possessing various biological functions as antioxidants to prevent cellular injury triggered by endogenous ROS. Vitamin E is a lipid-soluble molecule that acts within the lipid membrane to avoid the formation of hydroperoxides while Se is an important component of glutathione peroxidase to detoxify the hydrogen peroxide before damaging cellular molecules [13]. Ramirez-Bribiesca et al. [14] have also documented the beneficial effects of vitamin E and Se treatment on goats and their kids. The objective of our study was to explore the antioxidative effect of vitamin E and Se on pregnant and nonpregnant animals reared on saline land. According to our knowledge, this is the first study indicating the positive impact of parenteral induction of vitamin E and Se on the performance of animals reared on a saline environment.

## 2. Material and Methods

### 2.1. Ethical Statement

The experiment was approved by the Institutional Animal Care and Use Committee at the Nuclear Institute for Agriculture and Biology (NIAB), Faisalabad, Pakistan.

### 2.2. Study Plan and Animal Management

The research experiment was performed at the Biosaline Research Station (BSRS), Pakka Anna, which is located approximately 50 km in the southwest of Faisalabad, Punjab, Pakistan (31.233 latitude and 72.80 longitudes). The experiment lasted from September to mid-December 2019. BSRS comprises 400 hectares of the salt-affected land. The climate of the saline environment is semi-arid having rainfall of 200 mm, and the summer and winter temperature is 39°C and 14°C, respectively.

A total of thirty-six multiparous pregnant (*n* = 18) and nonpregnant (*n* = 18) healthy Beetal goats aged 2-4 years, weighing about 38-45 kg, were equally divided into pregnant (*n* = 9/9)/nonpregnant (*n* = 9/9) animals. The control group was given normal saline (NaCl 0.9%) subcutaneously, while the treated group was subjected to vitamin E (1000 mg/kg BW) and selenium (3 mg/50 kg BW), with the same parenteral route. Vitamin E and selenium (Myogaster, Inovet, Belgium) were procured from the local market of Faisalabad, Pakistan. The experiment of pregnant goats was initiated during the last month of pregnancy (120 days postbreeding) and lasted until the 15 days of post-parturition, while, for nonpregnant cyclic goats, it was started from day 0 and lasted on day 45 (50 days of post-kidding is considered day 0). Goats were allowed to graze on *Leptochloa fusca* (Kallar grass), *Acacia ampliceps*, *Atriplex lentiformis* (saltbush), and *Suaeda fruticosa* during the morning and evening schedule. Both groups of goats were provided concentrates (Table 1) with crude protein content above 11.5% at 200 g/day/animal and had given free access to saline drinking water. Blood sampling was done with 15 days interval except for progesterone detection in pregnant animals which was done till parturition. 6 mL of blood plasma was obtained into a vacutainer tube containing EDTA aseptically. Blood was brought to the laboratory and subjected to centrifugation at 3000 rpm at 4°C. After that, blood plasma was separated and kept at -80°C till further biochemical analysis [15].

At the start of the experiment, soil texture parameters (sand, silt, clay, salinity, pH, Na^+^, Ca^+++^, Mg^++^, Cl^−^, CO_3_^−2^, and HCO_3_^−^) from the field area were analyzed by using the method described in USDA Handbook 60 [16] as shown in Table 2 and water quality was tested according to the Boyd protocol [17] and is depicted in Table 3.

### 2.3. Biochemical Analysis

#### 2.3.1. Estimation of Enzymatic Antioxidant Activity

*(1) Superoxide Dismutase (SOD) Activity*. The obtained blood plasma was estimated for superoxide dismutase activity assayed by measuring its ability to inhibit the photochemical reduction of nitroblue tetrazolium (NBT) according to the method of Giannopolitis and Ries [18]. The reaction solution (3 mL) contained 50 *μ*M NBT, 1.3 *μ*M riboflavin, 13 mM methionine, 75 nM EDTA, 50 mM potassium phosphate buffer (pH 7.8), and 50 *μ*L blood plasma. The photoinduced reaction was performed in an aluminum foil-lined box fitted with a 15 W fluorescent lamp. The absorbance of the irradiated solution at 560 nm was determined with a spectrophotometer (Hitachi U-2800, Tokyo, Japan). One unit of SOD activity was defined as the amount of enzyme which caused 50% inhibition of photochemical reduction of NBT.

*(2) Catalase (CAT) Activity*. Catalase activity in blood plasma was assayed by a method described by Beers and Sizer [19]. For the measurement of CAT activity, the assay solution contained 50 mM phosphate buffer (pH 7.0), 59 mM H_2_O_2_, and 0.1 mL enzyme extract. The decrease in absorbance of the reaction solution at 240 nm was recorded after every 20 s. The absorbance change of 0.01 min^−1^ was defined as 1 U of CAT activity.

*(3) Peroxidase (POD) Activity*. Plasma POD activity was determined by using a method described by Change and Maehly [20] with few amendments. For the analysis of peroxidase activity, the assay solution contained distilled water (545 *μ*L), 200 mM phosphate buffer (pH 7.0), 200 mM guaiacol, 400 mM H_2_O_2_, and 15 *μ*L plasma sample. The reaction in the solution was started immediately after adding the sample. The increase in absorbance of the reaction solution at 470 nm was recorded after every 20 s. One unit of POD activity was defined as an absorbance change of 0.01 min^−1^.

#### 2.3.2. Nonenzymatic Antioxidants

*(1) Total Phenolic Content (TPC)*. Total phenolic contents in the blood plasma were assessed by the microcolorimetric method of Ainsworth and Gillespie [21] with some amendments in the Folin-Ciocalteu (F-C) reagent. A 100 *μ*L of blood plasma sample was mixed with 100 *μ*L of 10% (*v*/*v*) F-C reagent and vortexed thoroughly, and then, 800 *μ*L of 700 mM Na_2_CO_3_ was added. Samples were then incubated at room temperature for 1 h. Blank corrected absorbance of samples was measured at 765 nm. Phenolic content (gallic acid equivalents) of samples was determined using a linear regression equation.

*(2) Total Flavonoid Content (TFC)*. The total flavonoid content was determined according to the aluminum chloride colorimetric method [22]. Blood plasma was mixed with 0.1 mL of 10% aluminum chloride hexahydrate, 0.1 mL of 1 M potassium acetate, and 2.8 mL of deionized water. After the 40-minute incubation at room temperature, the absorbance of the reaction mixture was determined spectrophotometrically at 415 nm. Rutin was used as a standard (concentration range: 0.005 to 0.1 mg/mL), and the total flavonoid content was expressed as milligram RE per g of plasma. The absorbance at 415 nm is 14.171 crutin (mg/mL) + 0.0461 (*R*^2^ = 0.9991).

*(3) Ascorbic Acid*. To determine the ascorbic acid concentration, a simple method described by Hameed et al. [23] was used, which measures only reduced ascorbic acid. In brief, each molecule of vitamin C converts a molecule of DCIP into a molecule of DCIPH2, and conversion can be monitored as a decrease in the absorbance at 520 nm. A standard curve was prepared using a series of known ascorbic acid concentrations. A simple linear regression equation was calculated to find the ascorbate concentration in unknown samples.

#### 2.3.3. Hydrolytic Enzymes

*(1) Protease Activity*. The activity of protease was measured by the casein digestion assay according to the protocol by Drapeau [24]. In this method, one unit is the amount of enzyme, which releases acid-soluble fragments equivalent to 0.001 A280 per minute at 37°C and pH 7.8. Enzyme activity was expressed in blood plasma.

*(2) Esterase Activity*. The *α*- and *β*-esterases were estimated according to the method of Van Asperen [25] using *α*-naphthyl acetate and *β*-naphthyl acetate as substrates, respectively. The reaction mixture consisted of substrate solution (30 mM *α*- or *β*-naphthyl acetate, 1% acetone, and 0.04 M phosphate buffer (pH 7)) and blood plasma. The reaction mixture was incubated for 15 minutes at 27°C in the dark, and then, 1 mL of staining solution (1% Fast blue BB and 5% SDS mixed in a ratio of 2 : 5) was added and incubated for 20 min at 27°C in the dark. The amount of *α*- and *β*-naphthol produced was measured by recording the absorbance at 590 nm. Using the standard curve, enzyme activity was expressed as *α*- or *β*-naphthol produced in *μ*M/min^−1^/mL.

#### 2.3.4. Other Biochemical Parameters

*(1) Total Oxidant Status (TOS)*. Total oxidant status (TOS) was determined by referring to the method of Erel [26] in which the ferrous ion is oxidized into a ferric ion by oxidants present in the sample in an acidic medium and the ferric ion is measured with xylenol orange [23]. The assay mixture contained reagent R1, reagent R2, and sample extract. After 5 min, the absorption was measured at 560 nm by using a spectrophotometer. A standard curve was prepared using hydrogen peroxide. The results were expressed in *μ*M H_2_O_2_ equivalent per L.

*(2) Total Antioxidant Capacity (TAC)*. The reduction of 2,2-azino-bis(3-ethylbenzothiazoline-6-sulfonate) radical cation (ABTS^∙+^ that is blue-green) by antioxidants to its original colorless ABTS form is the basis of the ABTS assay. The ABTS^∙+^ is decolorized by antioxidants according to their antioxidant content [27]. The assay mixture contained reagent R1 (mixture of sodium acetate buffer solution and glacial acetic acid, pH 5.8), sample extract, and reagent R2 (mixture of sodium phosphate buffer solution, glacial acetic acid, hydrogen peroxide, and ABTS). The contents of the tubes were mixed and allowed to stand for 6 min. Absorbance was measured at 660 nm. Ascorbic acid was used to develop a calibration curve. The TAC values were expressed as millimolar ascorbic acid equivalent to l^−1^.

*(3) Malondialdehyde (MDA)*. The level of lipid peroxidation in the plasma was measured in terms of malondialdehyde (MDA, a product of lipid peroxidation) content determined by the thiobarbituric acid (TBA) reaction using the method of Health and Packer [28] with minor modifications as described by Dhindsa et al. [29]. A 25 *μ*L sample was homogenized in 0.1% TCA. The homogenate was centrifuged at 14,462 × *g* for 5 min. TBA was added to 1 m aliquot of the supernatant 20% TCA containing 0.05%. The mixture was heated at 95°C for 30 min and then quickly cooled in an ice bath. After centrifuging at 14,462 × *g* for 10 min, the absorbance of the supernatant at 532 nm was read and the value for the nonspecific absorption at 600 nm was subtracted. The MDA content was calculated by using an extinction coefficient of 155mM^−1^cm^−1^.

*(4) Protein Content*. Determination of quantitative protein in plasma was performed by the method of Bradford [30] in which 5 *μ*L of blood plasma sample and 0.1 N NaCl were mixed with 1.0 mL of Bradford dye and the mixture was allowed to stand for 5 min to form a protein-dye complex. Absorbance was calculated at 595 nm by using a spectrophotometer.

#### 2.3.5. Detection of Plasma Progesterone Levels

Plasma progesterone concentration was determined by a radioimmunoassay (Videogamma/Rack, automatic gamma counter, I'acn, Italy) according to the instructions of commercially available kits (DIAsource, ImmunoAssays S.A, Belgium). The sensitivity of the assay was 0.05 ng/mL, and the interassay and intra-assay coefficients of variation were 8.6 and 6.5%, respectively. The cross-reactivity with other steroids ranged from <0.01 to 15%.

#### 2.3.6. Measurement of Selenium in Blood Plasma

Inductively coupled plasma optical emission spectroscopy (ICP-OES) (Optima 2100-DV, PerkinElmer, Massachusetts, USA) was employed to determine the blood plasma concentration of selenium. Argon gas (99.99% pure, pressure 80-120 psi) creates the plasma flame at a flow rate of 0.80 L/min. Nitrogen gas (99.99% pure) is used as a purge gas with a flow rate of 0.5 L/min, and compressed air as a shear gas passed through the system at a flow rate of 2.0 L/min. A stable, high plasma temperature of about 7000-100000 K is usually generated. The ICP-OES uses a charge-coupled device which is a semiconductor photodetector to simultaneously analyze analytes.

The aspirated samples collided with ions and electrons. This process has broken samples into monatomic ions. The element gained energy during the collision process, became excited, and further relaxed. Upon particular wavelengths, the monatomic ions are split by optics and analyzed via a semiconductor photodetector—charged-coupled device (CCD). By the help of a standard calibration curve, the detection was obtained at the lowest levels of ppm. WinLab32 window software was used for analyzing selenium data.

#### 2.3.7. Statistical Analysis

All statistical analyses were performed using XL-STAT software version 2012.1.02 (Copyright Addinsoft 1995-2012) (http://www.xlstat.com). Descriptive statistics were applied to analyze and organize the resulting data. Data were analyzed using ANOVA with three replications. The significance of data was tested by analysis of variance and the Tukey (HSD) test at *P* < 0.01. Values presented in graphs are mean ± SE. GraphPad Prism (5.0) software was used to draw the graphs.

## 3. Results

### 3.1. Enzymatic Antioxidants

**Figure 1:**
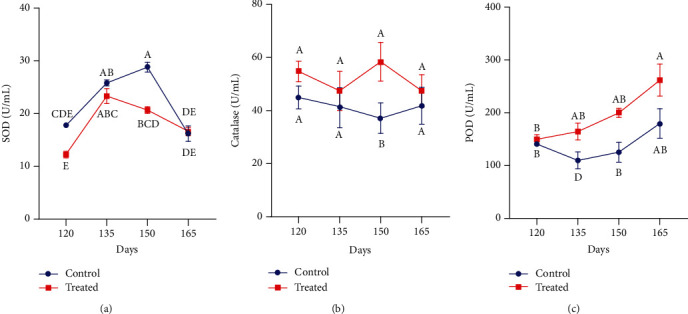
Effect of vitamin E and selenium supplementation on plasma (a) SOD, (b) catalase, and (c) POD activity of pregnant Beetal goats during the peripartum period at days 120, 135, 150, and 165 (3 times before parturition and one time after parturition). Values are presented as mean ± SE. Data points with different alphabets are statistically significantly different at *P* < 0.01.

The impact of Vit E and Se administration on superoxide dismutase, catalase, and peroxidase activities during the last phase of pregnancy in Beetal goats raised on saline land is shown in Figure 1. Plasma SOD activity increased before kidding and gradually reduced around parturition in the supplemented group. In non-supplemented pregnant goats, plasma SOD concentration was slightly increased and abruptly declined following parturition. Moreover, a significant effect of treatment was observed on plasma SOD activity at the time of expected parturition (150 days) (*P* < 0.01). In comparison with the control group, the plasma level of catalase and POD activity was recorded higher in animals receiving a subcutaneous injection of Vit E and Se. However, at parturition, catalase activity was significantly higher in the supplemented group as compared to the control group (*P* < 0.01).

**Figure 2:**
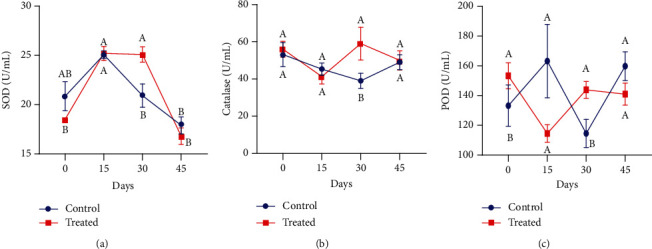
Impact of vitamin E and selenium administration on plasma (a) SOD, (b) catalase, and (c) POD activity of nonpregnant Beetal (cyclic) goats at days 0, 15, 30, and 45 (cyclic goats). Values are presented as mean ± SE. Data points with different alphabets are statistically significantly different at *P* < 0.01.

The effects of Vit E and Se supplementation on enzymatic antioxidants, i.e., SOD, catalase, and POD, in nonpregnant Beetal goats raised on saline land are shown in Figure 2. A significant effect of treatment was recorded on plasma SOD activity on day 30 (*P* < 0.01). Compared to the control group, the plasma level of catalase and POD activities was observed higher on day 30 of supplementation in animals receiving a subcutaneous injection of Vit E and Se. However, on day 45, POD activities were reduced in the treated group as compared to the control group.

### 3.2. Nonenzymatic Antioxidants

**Figure 3:**
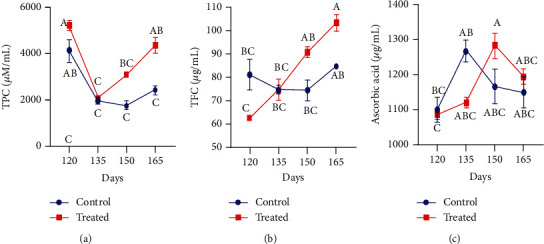
Effect of vitamin E and selenium induction on plasma concentration of (a) TPC, (b) TFC, and (c) ascorbic acid of pregnant Beetal goats on days 120, 135, 150, and 165 (3 times peripartum and one time postpartum). Values are presented as mean ± SE. Data points with different alphabets are statistically significantly different at *P* < 0.01.

Blood plasma of nonenzymatic antioxidant status in control and supplemented groups of pregnant goats is shown in Figure 3. The positive impact of Vit E and Se induction was observed in plasma activity of total phenolic contents, total flavonoid contents, and vitamin C in pregnant goats. The concentration of TPC and TFC in blood plasma was significantly higher around parturition in supplemented animals (*P* < 0.01). However, the level of Vit C also increased at parturition and declined afterwards in supplemented pregnant goats.

**Figure 4:**
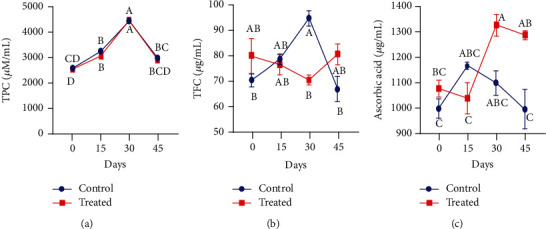
Effect of vitamin E and selenium on plasma concentration of (a) TPC, (b) TFC, and (c) ascorbic acid of nonpregnant Beetal (cyclic) goats at days 0, 15, 30, and 45 (cyclic goats). Values are presented as mean ± SE. Data points with different alphabets are statistically significantly different at *P* < 0.01.

The blood plasma level of nonpregnant Beetal goats has unveiled that total phenolic contents did not show any influence through antioxidant supplementation. However, irrespective of the group (either treated or control), the phenolic contents reached their maximum level on day 30 of the experiment (Figure 4(a)). Plasma total flavonoid contents were reduced in the treated group as compared to the control group following 15 days after the supplementation. At day 30 of supplementation, flavonoid contents significantly declined relative to the control group (*P* < 0.01). However, on day 45, their concentration gradually increased in the group receiving Vit E and Se treatment (*P* < 0.01) (Figure 4(b)). It is apparent from Figure 4(c) that Vit E and Se treatment significantly enhanced the ascorbic acid level from the start to the end of the experiment in the blood plasma of nonpregnant goats (*P* < 0.01). The ascorbic acid concentration recorded higher on day 30 in supplemented animals as compared to the nonsupplemented ones.

### 3.3. Hydrolytic Enzymes

**Figure 5:**
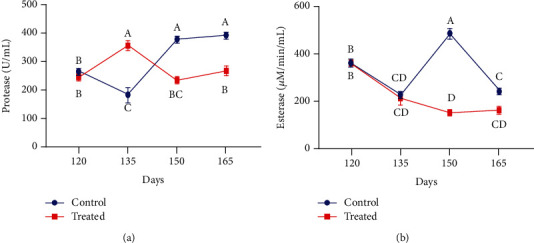
Effect of vitamin E and selenium administration on plasma concentration of (a) protease and (b) esterase activity of pregnant Beetal goats at days 120, 135, 150, and 165 (3 times before and one time after kidding). Values are presented as mean ± SE. Data points with different alphabets are statistically significantly different at *P* < 0.01.

The concentration of hydrolytic enzymes in the blood plasma of both control and treated groups of pregnant animals is presented in Figure 5. The protease and esterase levels were significantly lower at peripartum in supplemented pregnant goats than in the nonsupplemented ones (*P* < 0.01). After the 15 days of supplementation, protease level was found higher in the supplemented group than in the control group. However, around kidding, the activity of Vit E and Se significantly reduced the protease level. The plasma esterase concentration started to decline after 15 days to the end of the experiment.

**Figure 6:**
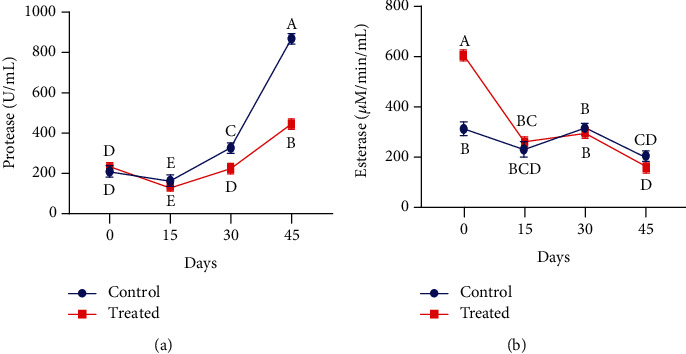
Influence of vitamin E and selenium induction on plasma concentration of (a) protease and (b) esterase activity of nonpregnant Beetal (cyclic) goats at days 0, 15, 30, and 45. Values are presented as mean ± SE. Data points with different alphabets are statistically significantly different at *P* < 0.01.

Plasma protease activity of nonpregnant Beetal goats was changed considerably from the start to the end of the study (Figure 6(a)). The level of protease enzyme was increased significantly on day 45 as compared to day 0 of treatment. Moreover, the protease concentration was significantly reduced in the treated group compared to the control group. However, a nonsignificant difference between control and treated Beetal goats was observed (*P* > 0.01). Following the injection of vitamin E and selenium, esterase activity was reduced significantly on day 15 in comparison with day 0 (Figure 6(b)). Interestingly, nonsignificant results were observed between both groups (*P* > 0.01), while significant results among both groups on different days were also noticed (*P* < 0.01).

### 3.4. Other Biochemical Parameters

**Figure 7:**
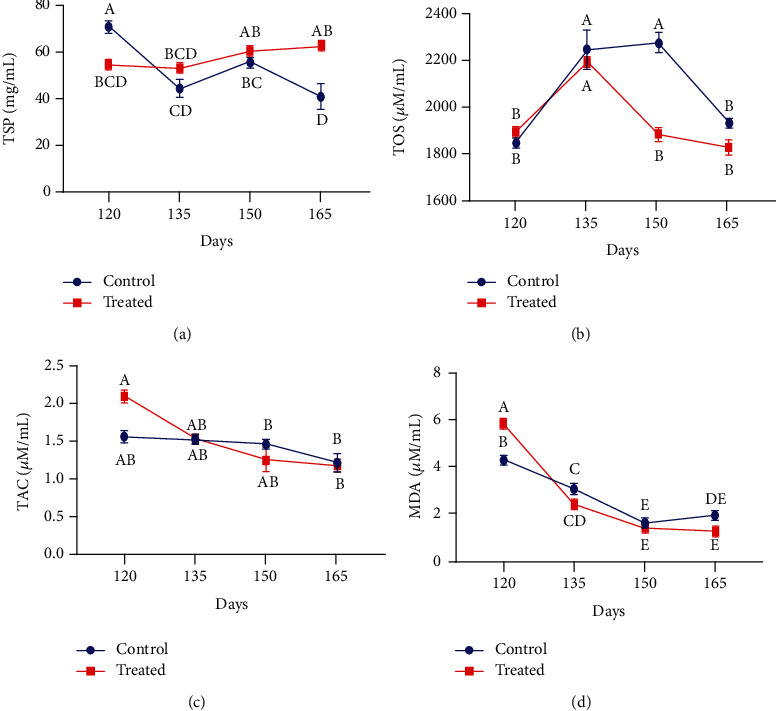
Impact of vitamin E and selenium supplementation on plasma of (a) total soluble protein, (b) total oxidant status, (c) total antioxidant capacity, and (d) malondialdehyde of pregnant Beetal goats during the peripartum period on days 120, 135, 150, and 165 (3 times peripartum and one time postpartum). Values are presented as mean ± SE. Data points with different alphabets are statistically significantly different at *P* < 0.01.

As depicted in Figure 7, a subcutaneous injection of Vit E and Se significantly affects plasma total soluble proteins (TSP) and total oxidant status (TOS) in pregnant animals. Blood plasma TSP level was significantly higher at postparturition in the supplemented group as compared with the control group (*P* < 0.01). Moreover, TSP concentration around parturition was observed numerically greater in the blood plasma of supplemented animals. Plasma oxidant status in treated pregnant goats at peripartum was significantly lower than that in control goats (*P* < 0.01). Regardless of the group (either supplemented or control), it was observed that MDA content in blood plasma at the last trimester of pregnancy declined as pregnancy progresses in both groups of animals. However, in the Vit E- and Se-supplemented group, the MDA concentration declined rapidly. In comparison with the control group, the promptly increased level of total antioxidant capacity was recorded in the supplemented group following parturition, although the treatment did not affect the TAC level before kidding.

**Figure 8:**
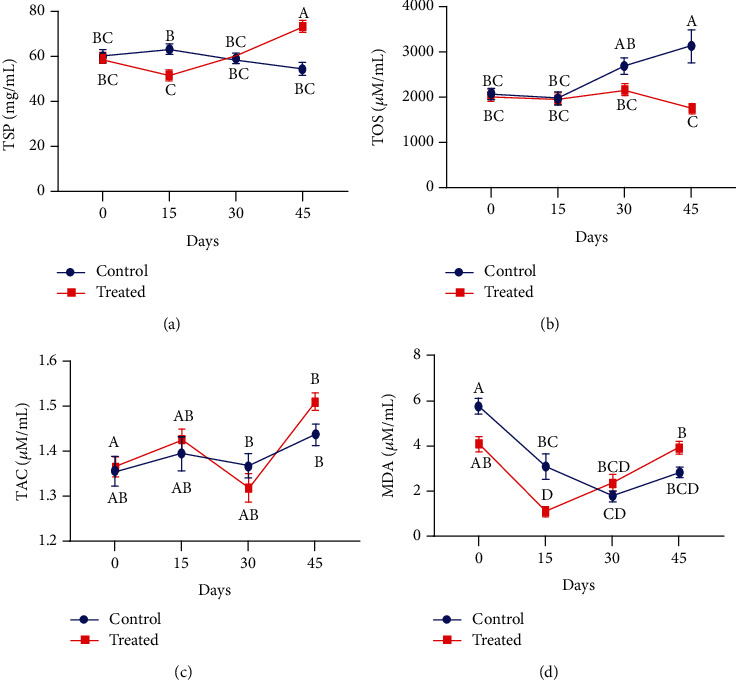
Effect of vitamin E and selenium induction on plasma concentration of (a) total soluble protein, (b) total oxidant status, (c) total antioxidant capacity, and (d) malondialdehyde of nonpregnant Beetal (cyclic) goats at days 0, 15, 30, and 45. Values are presented as mean ± SE. Data points with different alphabets are statistically significantly different at *P* < 0.01.

The supplemented and control groups of nonpregnant goats were given a comparable profile of plasma total proteins (TSP), total oxidant status (TOS), total antioxidant capacity (TAC), and malondialdehyde (MDA). Our findings have shown that treated goats had significantly higher levels of total soluble proteins at the end as compared to the start of the experiment. It was also observed that Vit E and Se supplementation significantly enhanced the soluble protein concentration in comparison with nonsupplemented animals (*P* < 0.01) (Figure 8(a)). The total oxidant status declined in the injected group on day 30 of treatment, and thereafter, it continued to decline significantly in comparison with the control group (*P* < 0.01). Some significant results were also recorded at some points between control and supplemented groups (*P* < 0.01) (Figure 8(b)). It is clear from Figure 8(c) that a subcutaneous injection of Vit E and Se increased the TAC levels at the end of the study. However, TAC concentration was increased on days 15 and 45 in comparison with the control group in Beetal goats (*P* > 0.01). Antioxidant supplementation showed a significant effect on plasma MDA concentration (*P* < 0.01) on day 15. However, on the next sampling, its little higher level was observed in the supplemented group in comparison to the control group (Figure 8(c)).

### 3.5. Selenium and Progesterone

**Figure 9:**
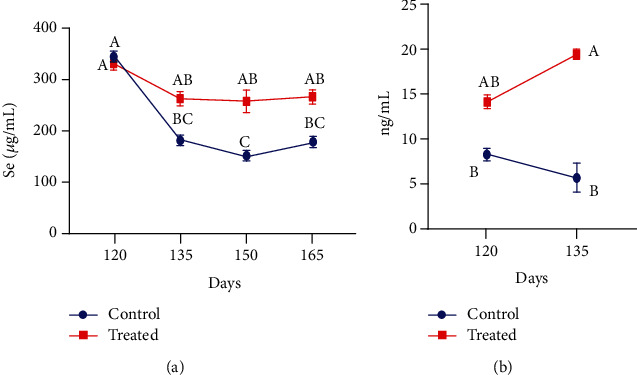
Effect of vitamin E and selenium supplementation on plasma concentration of (a) Se and (b) progesterone of pregnant Beetal goats during the peripartum period on days 120, 135, and 150 (3 times before and one time after parturition). For progesterone analysis, samples were only obtained on days 120 and 135 of pregnancy. Values are presented as mean ± SE. Data points with different alphabets are statistically significantly different at *P* < 0.01.

The blood plasma concentrations of Se (Figure 9(a)) and progesterone (Figure 9(b)) in the last trimester of pregnant supplemented and nonsupplemented goats are displayed in Figure 9. The results indicated that Se concentration only on day 150 was higher in the treated animals in comparison with control animals (*P* < 0.01). Moreover, the progesterone concentration was recorded higher only on day 135 in treated animals with Vit E and Se in comparison to nontreated animals.

### 3.6. Average Daily Weight Gain of Kids

**Figure 10:**
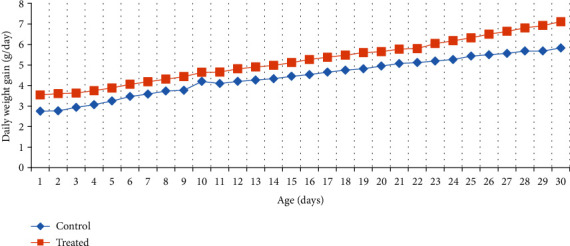
Effect of Vit E and Se on daily weight gain of goats' kids.

Figure 10 shows the daily weight gain of goat kids raised in a saline environment. The results revealed that animals subjected to Vit E and Se had increased daily weight gain with respect to the control group following thirty days of post-kidding.

### 3.7. Initial Birth Weight

**Figure 11:**
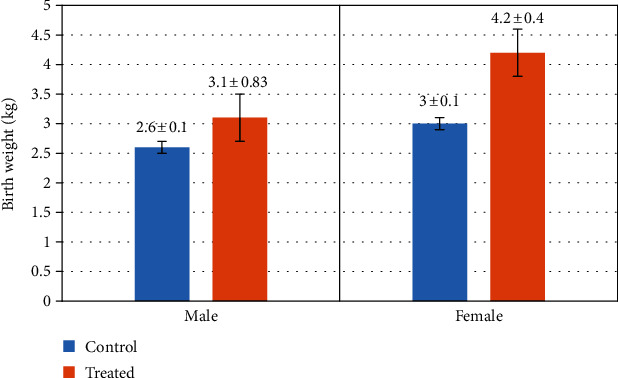
Effect of Vit E and Se on birth weight of goats' kids (male/female). The results are expressed as mean ± SD.

Figure 11 indicates the initial birth weight taken immediately after the parturition (on the day of kidding). It is clear from the results that the kids of treated goats had higher initial birth weight as compared to the control group.

### 3.8. Average Weight Gain

**Figure 12:**
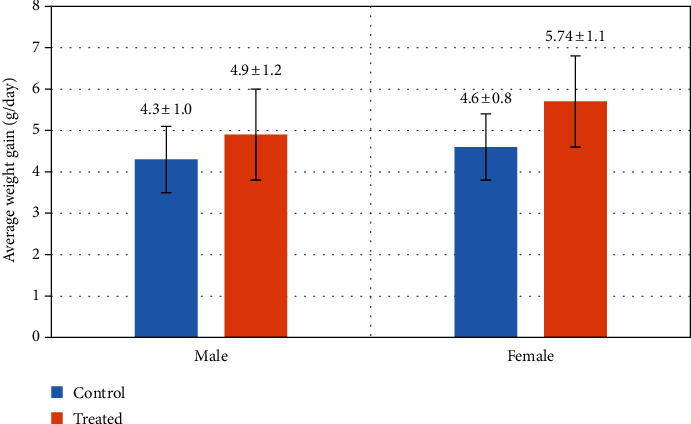
Effect of Vit E and Se on average weight gain of goats' kids (male/female). The results are expressed as mean ± SD.

Figure 12 shows the average weight gain of suckling kids of Beetal goats. The kids of goats who had received Vit E and Se injection had a higher average weight gain as compared to the kids of non-antioxidant-treated group.

### 3.9. Total Weight Gain

**Figure 13:**
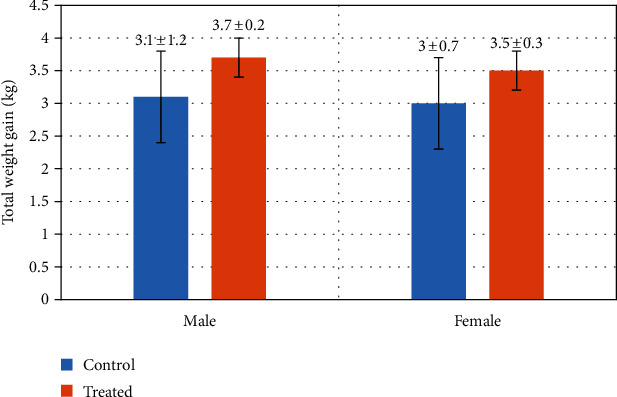
Effect of Vit E and Se on total weight gain of goats' kids (male/female). The results are expressed as mean ± SD.

The total weight gain of treated and control goat kids is shown in Figure 13. The suckling kids of the treated group had obtained higher total weight in comparison to kids of the control group.

### 3.10. Reproductive Performance

Table 4 illustrates the reproductive parameters of the control and treated groups of animals. The results showed that the survival rate of kids from the supplemented group (100%) was higher than that of kids from the control group (60%). The twining ratio was recorded to be 33% in the treated group, whereas no twining was recorded in control animals.

## 4. Discussion

Soil salinity is a dynamic problem, and its impact on land occurs since long. The livestock raised in such areas has to consume plants and water having a high level of salt content which may lead to disturbances in the redox status of animals [1]. Pregnant animals are more vulnerable to oxidative stress due to the alteration in redox balance which ultimately reduced yield [3]. Thus, our study was aimed at determining the antioxidant potential of Vit E and Se supplementation on saline environment-induced oxidative stress in pregnant and nonpregnant goats and its positive impact on reproductive and growth performance of sukcling kids.

The animal body is well equipped with a variety of protective mechanisms to neutralize the harmful effect of ROS. SOD stimulates the conversion of superoxide anion (O^−1^) to hydrogen peroxide (H_2_O_2_) and oxygen (O_2_). Furthermore, the catalase, another antioxidant enzyme, converts H_2_O_2_ into oxygen and water. Thus, the complete process of neutralization began with SOD [31]. It is clear from previous studies that the antioxidant system is suppressed in normal gestation while the saline environment exaggerates the problem [32]. In this current study, the activities of enzymatic antioxidants like SOD, catalase, and POD from blood plasma were determined after Vit E and Se supplementation in pregnant and nonpregnant goats. The higher concentration of catalase and POD indicated that parenteral administration of Vit E and Se improved the activity of some enzymatic antioxidants like catalase and POD. The protective effects of both Vit E and Se against reduced activities of enzymatic antioxidants caused by oxidative stress have already been reported in some other investigations. Amraoui et al. [33] described that Se efficiently reinforces the activity of cellular enzymatic antioxidants. Other supporting results in our study showed a beneficial effect on the same treatment [32]. Moreover, Vit E and Se administration upregulates and activates some antioxidant enzymes [34] and thereby provides beneficial effects. In our previous study, conducted on normal land, antioxidant supplementation significantly increased the level of antioxidant enzymes [35].

Flavonoids and phenolics are potent antioxidant compounds in animals and ingested in a significant amount from plant sources through grazing. The flavonoids protect against oxidation in the biological system via different mechanisms, including suppressing the ROS formation by inhibition of enzymes that contribute to the free radical production, upregulating/protecting antioxidant defense, or directly scavenging reactive oxygen species [36]. Our results confirmed that antioxidant supplementation (Vit E and Se) enhanced the level of flavonoids and phenolics in the blood plasma of pregnant goats while nonsignificant results were observed in nonpregnant animals. It could be suggested from the obtained results that the saline environment and pregnancy enhanced different stresses especially oxidative stress in animals which was attenuated with the injectable form of Vit E and Se by removing the deleterious effects of ROS. To the best of our knowledge, this is the first study in which flavonoids and phenolic contents were measured following Vit E and Se administration.

Vit C is considered an important constituent of the antioxidant defense system, enabling the minimization of the damaging effects of salinity-induced oxidative stress [37]. According to earlier studies, the level of Vit C in blood plasma during normal pregnancy was decreased as the pregnancy advances which makes animals susceptible to oxidative damage [38]. Our results are in accordance with the results of Rao et al. who also observed decreased Vit C concentration in typical gestation [39]. In our study, increased Vit C level was recorded in treated animals, suggesting that antioxidant supplementation promoted Vit C and thereby provided protective effects as a free radical scavenger. Vit C is a water-soluble vitamin and reported as the first line of antioxidant defense against the detrimental form of ROS in blood plasma. ne explanation is that Vit E being the lipid-soluble antioxidant and Se, as a cofactor of the GPX enzyme prevents the overproduction of cellular oxidants; thereby, their levels are reduced in plasma concentration. In this way, supplementation protects the pregnant goats from oxidant insult due to the saline environment and pregnancy.

It has been demonstrated from previous studies that accelerated production of ROS and other free radicals or oxidants can be a source of serious injury to cellular organelles and other biomolecules. These damaged biomolecules show a pool of worthless cellular debris that can cause various immunologic and metabolic problems. Thus, all cells must have both membrane-bound and soluble hydrolytic/proteolytic enzymes for the elimination or reutilization of such debris. This proteolytic system is of great importance and behaves as a secondary antioxidant defense system where the level of primary antioxidants is insufficient to protect against a high degree of oxidative stress [40]. In this study, protease and esterase activity was significantly higher in supplemented animals; therefore, it shows the protective effect of Vit E and Se against oxidative stress at the cellular level.

Pregnancy is considered the intense protein or metabolic alteration period as reported in previous studies [41, 42]. The significant decline in blood plasma or serum proteins during the peripartum stage has been described in different animal species including goat and sheep [41–45]. The reduction of maternal blood plasma proteins in the last trimester of pregnancy seems to be due to the high demand for amino acids transferring from the mother to the fetus for numerous protein syntheses necessary for growing fetuses [46]. Therefore, this study indicated that the total blood plasma protein level was reduced in the control group as compared to the supplemented group. Parenteral administration of Vit E and Se increased blood plasma protein concentration during the last trimester of pregnancy which showed that a higher demand for proteins is involved in a developing fetus. These results are in line with the findings of other studies conducted on pregnant ewes, buffaloes, and dairy cows, respectively [47–49]. The higher level of plasma proteins in the supplemented group considering the important role of Se element in protein synthesis acts as a component of various selenoproteins which was previously studied [50, 51]. Selenocysteine-based selenoproteins exhibit a significant role in different body functions like antioxidant defense and reproductive function [52].

The total antioxidant capacity (TAC) in blood plasma was measured through the ABTS assay. The ABTS method is recommended to determine the relative capacity of total antioxidants in plasma to scavenge ABTS as compared to the Trolox standard [53]. The current finding showed that initially TAC level abruptly declined as parturition approaches, but supplementation of Vit E and Se inhibits a further decrease irrespective of the group in which TAC level declined around parturition, while in the case of nonpregnant goats, the supplementation inhibited the reduction of TAC level as the experiment advances. Our results are in accordance with previous studies where blood plasma TAC concentration was noticed higher in sheep and goats following the induction of Vit E and Se treatment [54–56]. Chauhan et al. [34] also reported the elevated level of TAC in blood plasma after the same antioxidant injection.

Lipids or polyunsaturated fatty acids are most susceptible to highly reactive radicals or nonradicals and trigger the process of lipid peroxidation. This process produces different substances which are mainly used as markers for the determination of lipid peroxidation. Among them, malondialdehyde (MDA), a product of lipid peroxidation, has been extensively used for a long time as a suitable biomarker to study the evidence of oxidative stress occurrences [57]. The results of this study indicated that parenteral administration of Vit E and Se protected both pregnant and nonpregnant goats from lipid peroxidation and decreased the MDA concentration in blood plasma during the last phase of gestation. These results are in accordance with previous experiments which also proved that ewes with a low concentration of Vit E and Se showed elevated MDA levels [58, 59]. Vit E and Se play a synergistic role in protecting the cells or cellular organelles from the harmful effects of lipid peroxidation. Vit E is found in different cellular membranes and prevents the peroxidation process, while the Se element plays its function in the cell cytoplasm by destroying peroxides [60].

It has been reported that small ruminants have the highest blood plasma progesterone concentration during 60-140 days of gestation. As the pregnancy advances to parturition, progesterone level in blood plasma decline to 1 ng/mL. ROS displays an important role in animal reproduction derived from the corpus luteum; once the level of ROS is increased than normal, it may prevent the progesterone synthesis formation by cytochrome P450 inhibition and intracellular mitochondrial cholesterol transport [61]. In this research experiment, a higher level of blood plasma progesterone concentration was observed in supplemented pregnant goats indicating a strong correlation with progesterone and depicts an important impact on pregnancy. Kamada and Hodate also reported a similar finding and reported a higher level of progesterone in animals supplemented with Se. Our results are in parallel with the previous finding in dairy cows that were subcutaneously administrated Vit E and Se and observed an increased level of progesterone [62]. Progesterone is a steroid hormone that is responsible for embryonic growth and survival by stimulating and maintaining the functions of the endometrium, and abrupt reduction may lead to abortion in animals [63]. In this experiment, 100% fetus survival rate was observed in the supplemented group while 40% of animals showed abortion in the control group during the trimester of pregnancy. Based on obtained results, it might be suggested that control group animals experienced a higher level of oxidative stress due to the saline environment exaggerating the problem that leads to abortion, while antioxidant supplementation stabilized the progesterone level and prevented abortion.

Se is a trace element and known to exist as an essential component in many selenoproteins and the primary enzymatic antioxidant GSH-Px [51]. Se is also found in different amino acids such as selenomethionine/selenocysteine that are involved in various functions, mainly antioxidant defense system, thyroid hormone synthesis, and reproduction [52]. In the last trimester of pregnancy, the demand for Se increased for dam as well as for growing fetuses. Therefore, the level of Se decreased as the pregnancy advances leading to oxidative stress, which is reported in different earlier studies [14]. The current study showed that Vit E and Se increased the level of Se in the blood plasma of treated goats. The results suggested that the injected dose of Se is effective to maintain the Se level of pregnant goats. The findings of this experiment are in line with the previous report where Vit E and Se increased blood plasma concentration of Se in goats reared in selenium-deficient soil [14]. Our results are in agreement with the previous studies in which combination of Vit E and Se increased the level of plasma Se and improved the reproductive performance in goats [64, 65].

It was noticed in our results that the survival rate was higher in the supplemented group of animals. Similarly, induction of Vit E and Se showed a significant effect on initial birth weight, average daily gain, and total weight gain of suckling kids of Beetal goats. Previous studies on ewes reported higher birth weight as well as higher average daily weight gain in the antioxidant-supplemented group. The improved productivity of ewes and goats was consistent with the earlier experiments in terms of survival rate and daily weight gain of neonates [66, 67]. Lambs raised by ewes treated with Vit E and Se in the last phase of gestation presented better weight gain than those not receiving any treatment [56]. A possible reason is due to the induction of Vit E and Se in the last trimester of pregnancy, increasing the transfer of Se to the growing fetus and enhancing its concentration in the colostrum as well as milk secretion, which improved feed intake efficiency and average daily weight gain [68]. Previous studies conducted on cattle and sows reported that the administration of Vit E and Se in late pregnancy enhances the survival rate of the fetus [69]. Supplementation of Vit E and Se in the late phase of pregnancy can decrease the utilization of energy to replace or repair damaged cells during gestation, and sufficient energy will be available for the developing fetus [70]. Oxidative stress experienced in the last trimester of pregnancy could lead to several reproductive disorders [70]. Gur et al. [71] reported that the administration of powerful antioxidants in late gestation could protect both dam and fetus against damaging outcomes of oxidative stress.

## 5. Conclusion

In conclusion, parenteral administration of vitamin E (1000 mg/kg BW) and Se (03 mg/50 kg BW) ameliorated saline environment-induced oxidative stress in late gestation by enhancing the antioxidant indices, improved the reproductive performance, and showed positive effects on growth performance of suckling kids. Moreover, further studies should be conducted on a saline environment by increasing the number of animals and different strategies of antioxidant supplementation along with antioxidant status of suckling kids.

## Figures and Tables

**Table 1:** Chemical composition of concentrates. The crude protein content was above 11.5%.

Feed ingredients	Percentage
Wheat straw	20
Wheat bran	15
Wheat grains	18
Mung bean grains	20
Mung bean straw	20
Linseed	5
Dicalcium phosphate	2
Total	100

**Table 2:** Soil texture analysis.

Soil depth (cm)	Sand (%)	Silt (%)	Clay (%)	Salinity (dSm^−1^)	pH	Na^+^(meL^−1^)	Ca^++^+Mg^++^ (meL^−1^)	Cl^−^ (meL^−1^)	CO_3_^−2^(meL^−1^)	HCO_3_^−^(meL^−1^)
0-15	54.24	8	37.76	6.7	8.58	59.1	4.8	24	1.2	17.4
15-30	54.24	8	37.76	8	8.68	98	2.4	30	1.2	20.7
30-60	54.24	8	37.76	5	8.54	40.5	1.8	13.8	0.9	14.1
60-90	54.24	10	37.76	5.1	8.53	47.7	2.4	15.6	0.9	11.1
90-120	44.24	18.72	37.04	5.3	8.87	50.5	1.2	36	1.5	12
120-150	50.24	14.72	35.04	12.7	8.84	108	2.4	42.6	1.5	12.6
150-180	54.24	8.72	37.04	13.4	8.86	129	1.8	43.5	2.1	16.3

Values are mean of four composition soil samples at each depth. Soil texture data from the field area was analyzed using the method from the USDA Handbook 60 [17]. Abbreviations: Na^+^: sodium ion; Ca^++^: calcium ion; Mg^++^: magnesium ion; Cl^−^: chlorine ion; CO_3_^−2^: carbonate ion; HCO_3_: bicarbonate ion; cm: centimeter; dSm^−1^: deciSiemens per meter; meL^−1^: millimole per liter.

**Table 3:** Water quality analysis parameters.

Electrical conductivity (dSm^−1^)	6.65
pH	7.6
Chloride (meL^−1^)	34.25
Residual sodium carbonate (meL^−1^)	17.15
Sodium absorption ratio	34.25

Water quality parameters were measured using the earlier Boyd protocol [18].

**Table 4:** Effect of vitamin E and selenium supplementation on reproductive parameters in Beetal goats during the postkidding period of 30 days.

Group	Twining (%)	Survival rate (%)	Male (%)	Female (%)	M/F (mean ± SD)/BW (kg)	M/F (mean ± SD)/ADG (g)	M/F (mean ± SD)/TWG (kg)
Control (*n* = 13)	0	62	40	60	2.6 ± 0.1/3 ± 0.1	4.3 ± 1.0/4.6 ± 0.8	3.1 ± 1.2/3 ± 0.7
Treated (*n* = 13)	25	100	62	38	3.1 ± 0.8/4.2 ± 0.4	4.9 ± 1.2/5.74 ± 1.1	3.7 ± 0.2/3.5 ± 0.3

Abbreviations: M: male; F: female; BW: birth weight; ADG: average daily weight; TWG: total weight gain.

## Data Availability

All the data used in this study in the form of tables and graphs will be available.
